# Early Childhood Assessments of Community Pediatric Professionals Predict Autism Spectrum and Attention Deficit Hyperactivity Problems

**DOI:** 10.1007/s10802-012-9653-4

**Published:** 2012-06-12

**Authors:** Merlijne Jaspers, Andrea F. de Winter, Jan K. Buitelaar, Frank C. Verhulst, Sijmen A. Reijneveld, Catharina A. Hartman

**Affiliations:** 1 Department of Health Sciences, University Medical Center Groningen, University of Groningen, P.O. Box 196, 9700 AD Groningen, The Netherlands; 2 Department of Psychiatry, Radboud University Nijmegen Medical Centre, Academic Center of Child and Adolescent Psychiatry, Nijmegen, The Netherlands; 3 Department of Child and Adolescent Psychiatry, Erasmus MC-Sophia Children’s Hospital, Rotterdam, The Netherlands; 4 Department of Psychiatry, University Medical Center Groningen, University of Groningen, Groningen, The Netherlands

**Keywords:** Autism, ADHD, Risk factors, Longitudinal studies, Child development

## Abstract

For clinically referred children with Autism Spectrum Disorder (ASD) or Attention Deficit/Hyperactivity Disorder (ADHD) several early indicators have been described. However, knowledge is lacking on early markers of less severe variants of ASD and ADHD from the general population. The aim of the present study is to identify early indicators of high risk groups for ASD and ADHD problems based on routine data from community pediatric services between infancy and age four. Data are from 1,816 participants who take part in Tracking Adolescents’ Individual Lives Survey (TRAILS), a longitudinal study. Information on early developmental factors was extracted from charts of routine Preventive Child Healthcare (PCH) visits. To assess ASD and ADHD problems, respectively, we used the Children’s Social Behavior Questionnaire (CSBQ) and the Child Behavior Checklist (CBCL), filled out by parents three times between the ages of 11 and 17. Note that these are parent ratings and not diagnostic instruments performed by trained clinicians. Male gender, low birth weight, low level of education of the mother, social, behavioral, language, psychomotor and eating problems significantly predicted ASD problems (odds ratios (OR) between 1.34 and 2.41). ADHD problems were also predicted by male gender and low level of education of the mother and by maternal smoking during pregnancy, good gross motor skills in first year*,* early attention and hyperactivity problems, and absence of parent-reported positive behavior (ORs between 1.36 and 1.74). Routine data on early childhood from PCH services are predictive for ASD and ADHD problems in adolescents in the general population. The PCH services are a useful setting to identify high risk groups, and to monitor them subsequently.

## Introduction

Autistic spectrum disorders (ASD) and Attention Deficit/Hyperactivity Disorder (ADHD) are neurodevelopmental disorders with an onset early in life, with a prevalence of around 1 % and 5 %, respectively (St Pourcain et al. 2011; Rommelse et al. 2011). ASD and ADHD often co-exist. Estimates on overlap in children with ASD range from 28 % to 53 %; ASD problems in children diagnosed with ADHD are also very common, although precise estimates are lacking (Hattori et al. 2006; Nijmeijer et al. 2010; St Pourcain et al. 2011; Reiersen et al. 2007; Rommelse et al. 2011; Ronald et al. 2010; Simonoff et al. 2008). A recent study showed that a complex longitudinal pattern exists between social-communication and hyperactivity-inattentive traits. The majority of children with persistent hyperactive-inattentive symptoms also showed persistent social-communication deficits but not vice versa (St Pourcain et al. 2011). Recently the field has broadened its focus so that it now views ASD and ADHD as constituting the very severe end of continuous distributions in the general population (Constantino and Todd, 2005; Levy et al. 1997; Stilp et al. 2010). Less severe variants of ASD and ADHD below the threshold for a diagnosis seem common, as shown by both family studies and studies in the general population (Constantino and Todd, 2005; Levy et al. 1997; Losh et al. 2009; Thapar et al. 2001). We further denote these as ASD and ADHD problems. Individuals with these subthreshold problems have similar but milder impairments in social functioning, communication, and information processing relative to children with a clinical diagnosis (Constantino and Todd, 2005; Hartman et al. 2012; Losh et al. 2009; Piven, 2002; Thapar et al. 2001). Thus, even at subthreshold level, problems may be an important burden for the children themselves, their parents, and others in their environment which may be alleviated by treatment.

Research has shown that early identification of ASD and ADHD, if followed by adequate intervention, may improve prognosis (Rogers and Vismara, 2008; Salmeron, 2009; Yirmiya and Charman, 2010; Zwaigenbaum et al. 2009). It seems important to identify, monitor and, if necessary, intervene in these developmental problems as early as possible. Community pediatric services, such as those in the USA and Europe, are in a unique position for early identification of developmental problems in children. Early indicators are needed to monitor high risk groups (who very likely will develop additional problems or impairments), but there is a dearth of evidence on these in the general population. For clinically referred children with ASD or ADHD, however, several early indicators have been reported in the literature; these concern pre- and perinatal factors (Arnoudse-Moens et al. 2009; Courchesne, 2004; Kolevzon et al. 2007; Mouridsen et al. 2008; Ploeger et al. 2010; Schendel et al. 2009; Smidts and Oosterlaan, 2007), early behaviors and emotions (Hartley et al. 2008; Hirshfeld-Becker et al. 2002; Loe and Feldman, 2007; Ozonoff et al. 2010; Smidts and Oosterlaan, 2007; Zwaigenbaum et al. 2005), motor and speech problems (Hagberg et al. 2010; Landa and Garrett-Mayer, 2006; Loe et al. 2008) and on atypicalities in regulation functions including sleeping and eating (Goodlin-Jones et al. 2008; Sung et al. 2008; Werner and Dawson, 2005). The age of observation of these indicators varies, and fluctuates between 6 months and three-and-a-half years (Zwaigenbaum et al. 2005; Zwaigenbaum et al. 2009).

The aim of the present study is to identify early indicators of high risk groups for ASD and ADHD problems based on routine data from community pediatric services between infancy and age four. The present study is complementary to previous prospective work in that it is the first to focus on the general community by using data from routine Preventive Child Healthcare (PCH) measurements, and from birth up to age four. The presence of ASD and ADHD problems was assessed during (pre-) adolescence. Note that previous studies on early indicators were focused on either ASD or ADHD. Given frequent co-occurrence (Hattori et al. 2006; Nijmeijer et al. 2010; Reiersen et al. 2007; Rommelse et al. 2011; Simonoff et al. 2008) and evidence of overlapping early indicators when the separate literatures on ADHD and ASD are brought together, their simultaneous study may further our insight into which indicators are unique to these disorders and which might be viewed as generic indicators for both. For the purpose of early detection and treatment, this is highly relevant.

## Methods

The TRacking Adolescents’ Individual Lives Survey (TRAILS) is a prospective cohort study among Dutch (pre-)adolescents, beginning at 10–12 years of age, that focuses on adolescent psychosocial development and mental health in the general population (de Winter et al. 2005; Huisman et al. 2008). Of all the pre-adolescents approached for enrollment in the study (n = 3,145), 6.7 % were excluded in case of mental (IQ < 60 based on school information) or physical incapability, or if no Dutch-speaking parent or parent surrogate was available. Seventy-six percent of the remaining 2935 pre-adolescents and their parents agreed to participate. The present study involves data from the first (n = 2,230; mean age = 11,09; SD = 0.56; starting March 2001), second (n = 2,149, mean age = 13.55 SD = 0.54) and third waves (n = 1,819, mean age = 16.26 SD = 0.73), and also involves data from the PCH files. Parents of 2,139 (96 %) children gave written informed consent to retrieve their child’s file from the PCH at T1. Out of these, 84.9 % could be traced (n = 1,816 PCH files). Children with and without a PCH file differed in parent-rated ASD (M = 5.45, SE = 0.13 for the retrieved and M = 7.25, SE = 0.36 for the non-retrieved, *P* < 0.001, i.e. higher scores among the non-retrieved) and ADHD problems (M = 3.83, SE = 0.08 vs M = 4.41, SE = 0.19, respectively, *P* < 0.01) at T1. Both for ASD and ADHD problems, higher scores indicate more symptoms. The TRAILS study was approved by the national ethical committee ‘Central Committee on Research involving Human Subjects’.

### The Outcome Measures: ASD and ADHD Problems

Parents filled out the Children’s Social Behavior Questionnaire (CSBQ) and the Child Behavior Checklist (CBCL) at all three measurement waves. The CSBQ is a validated questionnaire for child social problems typically seen in children with ASD, especially in its milder forms, for ages 4–18 (Hartman et al. 2006). The sum of the subscales of social interest, social understanding, stereotyped behavior, and resistance to change was used, which captures the core symptoms of ASD and consists of 30 items (‘t Hart-Kerkhoffs et al. 2009). The CBCL is an internationally validated questionnaire for child emotional and behavioral problems for ages 4–18 and consists of 118 items (Achenbach et al. 2003). From this questionnaire we used the DSM-IV-oriented attention-hyperactivity problem scale as outcome measure. This scale consists of seven items.

### Early Indicators

In the Netherlands, PCH services provide health and developmental monitoring for all Dutch children from birth until age 19; the participation rate is over 90 %. The information on indicators had been collected by community physicians and nurses (PCH professionals) as part of the routine procedure of the PCH. These assessments included a general physical examination, standardized screening procedures and an interview with parents concerning health status and developmental (physical, emotional and behavioral) problems, which were all documented in the PCH file. An assessment takes approximately 10 to 15 min. In accordance with the literature we selected all potentially relevant predictors: pre- and perinatal factors, BMI and head circumference growth curves, psychomotor factors, early childhood behaviors, and sociodemographic variables from the PCH file*.*

### Pre- and Perinatal Variables

Pre- and perinatal variables concerned maternal smoking and alcohol use during pregnancy, low birth weight (registered in grams) and birth defects as provided by the obstetrician or midwife. Maternal smoking and alcohol use were assessed as: “Did the mother smoke during pregnancy?” and “Did the mother use alcohol during pregnancy?” These questions were part of the PCH files, but were also part of the T1 interview in TRAILS (when a child was age 11 years). If either answer was “yes,” we defined a score as indicative of smoking or alcohol use, respectively. The obstetrician or midwife could report yes or no, and in case of yes the amount per week or day. Often only yes or no was filled out, so more detailed information on quantity could not be retrieved. Low birth weight was defined as <2500 grams, which is the standard clinical cut-off point. Birth defects included limb deformities, craniofacial malformations and anomalies in organs, which are often seen in children with a clinical diagnosis of autism (Ploeger et al. 2010). Respondents received a “yes” if any of these were present and a “no” if none were present.

### BMI and Head Circumference Increase

BMI at age 2, 3 and 4 years (extracted from weight and height in the PCH files) was categorized into: low BMI, intermediate BMI and high BMI; gender and age-specific cut-offs were used (Cole et al. 2000; Cole et al. 2007). Head circumference increase, from birth until age six months and from age 6 months until 12 months, was standardized into Mean Z-scores (Courchesne et al. 2003), and then dichotomized into the highest 10 % versus the rest.

### Psychomotor Development

Psychomotor development from birth to age four was assessed by three indices. The first was the Van Wiechen Scheme designed for children aged between 1 month and 15 months, which is the Dutch equivalent of the Bayley scales (Jacobusse et al. 2006). The PCH professional assigns a pass or fail score to each indicator for a given child. Indicators are divided into three different subcategories: gross motor skills (16 items), fine motor skills and adaptation (11 items), and communication and social behavior (10 items); each is targeted at children of a certain age. Items within these three subcategories are summed to provide subscales.

The second index concerned psychomotor development from the age of 18 months until four years old, with six measurements in this period, and was assessed as: “Does the child have any problems with motor skills and/or with speech and language” with both reported by either a “yes” or “no”. Motor skills, and speech and language, were each added up, respectively, and then dichotomized to a “yes” if any of these were present during these six occasions, and a “no” if none were present.

The third index concerned neurological problems observed during physical examination of the child, conducted six times between the ages of 18 months and 4 years old. If on any occasion a problem was present, this index was coded as “yes”, and as “no” if none were present.

### Early Childhood Behavior

Early childhood behavior refers to behavioral features which were the most striking in the child and were recorded between ages 1.5 to 4 years, for which we distinguished “sleeping, eating and emotional and behavioral problems.” During PCH visits, the PCH professional inquired into these problems as: “How is your child doing as far as eating is concerned?” and “How is your child doing as far as sleeping is concerned?” Difficulties such as “has problems falling asleep”, “wakes up and can’t sleep afterwards”, “eats with much problems” or “eats very little” were reported. Descriptions of these behaviors were categorized as “yes,” in case of problems, and “no” or “missing.” The responses to the six times that these behaviors were probed were added up, and then dichotomized to a “yes,” if any of these were present, and a “no,” if none were present. “Emotional and behavioral problems” were collected from three open questions in the PCH files, that is, “How does the child play,” “How is the child’s behavior?” and “How is the child’s social behavior?” The community physician or the nurse could report difficulties here such as “overactive,” “shy,” “anxious” or “aggressive,” but also strengths such as “social” (see Table 1). These PCH registered descriptions were categorized as social problems in behavior, attention hyperactivity problems, internalizing problems, externalizing problems or absence of parent-reported positive behavior and then dichotomized to a “yes” if any of these were present during these six occasions, and a “no” if none were present.

**Table 1:** Description of behavior of children by PCH professionals divided into five categories

Social problems in behavior	Attention/hyperactivity problems	Emotional problems	Behavioral problems	Positive behavior
Cannot be made enthusiastic about things;	Boisterous;	Anxious;	Aggressive;	Affectionate;
Cannot stand change;	Concentration problems;	Clings to mother;	Argues a lot;	Attentive;
Child with a “manual”;	Difficulty concentrating during play;	Cries easily;	Argumentative;	Cheerful;
Difficulty getting used to others;	Distracted/hyperactive;	Easily frightened;	Bold;	Confident;
Difficulties getting used to new situations;	Dreamy;	Fear of failure;	Bites others;	Cooperative;
Does not like many things happening simultaneously;	Easily bored;	Fear of abandonment;	Behavioral problems;	Cool, composed;
Does not respond to initiatives by others;	Impulsive;	Insecure;	Defiant;	Cuddly;
Easily manipulated;	Jumps from one thing to another;	Introvert;	Demands attention in a negative way;	Curious;
Hits him/herself;	Noisy;	Nervous;	Disobedient;	Cute;
Head banging;	Pushes the limits;	Over-sensitive;	Flings toys;	Eager to learn;
Immature;	Reacts to everything and everyone;	Over-submissive;	Gets “beside him/herself”;	Easy;
Inappropriate play;	Restless;	Panics easily;	Harassing;	Enthusiastic;
Social interaction does not extend beyond caretakers;	Sees no danger;	Quiet;	Headstrong;	Flexible;
Needs very strict routines;	Talks excessively;	Separation anxiety;	Hits others;	Focused;
Peculiar child;	Turbulent;	Silent;	Impatient;	Helpful;
Resists new things;	Very active;	Shy;	Impertinent;	Independent;
Sometimes hard to connect with;	Volatile;	Timid;	Imposing;	Interested;
Withdrawn	Wild;	Worried	Kicks others;	Kind;
	Short attention span		Manipulative;	Lively;
			Moody;	Looks after others;
			Nagging;	Nice;
			Oppositional;	Open;
			Overly present;	Patient;
			Provocative;	Picks up on things easily;
			Quickly becomes angry;	Polite;
			Rebellious;	Rich imagination;
			Stubborn;	Social;
			Temper tantrums;	Sociable;
			Whining	Spontaneous;
				Sweet;
				Takes the initiative

### Sociodemographic Variables

Sociodemographic variables refer to the highest educational level of the father and the highest educational level of the mother. We have distinguished two groups here: low (lower tracks of secondary education or less), and middle and high (higher tracks of secondary education, and university degree or more) educational levels, respectively.

### Statistical Analysis

Prior to analyses, both outcome measures (CSBQ and CBCL) were dichotomized. To determine the optimal cut-off point for dichotomization for both questionnaires, we plotted the cumulative score distribution of decentile scores (x-axis) versus raw scores (y-axis). All graphs showed steep increases in problem behavior with a consistent kink at the 80^th^ percentile. From that point onwards children began to have substantially more problems. For the CSBQ, the kink at the 80^th^ percentile was at 9 for all three measurements; for the ADHD scale of the CBCL it was 7. We defined a score above the 80th percentile at least twice (out of three measurements) as indicative of the presence of ASD and ADHD problems. Note that these questionnaires were not diagnostic tools performed by trained clinicians.

Subsequently we calculated descriptive statistics for all predictor variables and performed logistic regression analysis to examine the influence of early development on ASD and ADHD behaviors. Based on the results of the univariate analyses, we considered variables associated with the outcomes at *p* < 0.2 to be candidates for multiple logistic regression analyses. Variables were entered into a backward stepwise logistic regression procedure, generating a subset of independent predictors for the two outcome measures. As a final step, we investigated whether our final multivariate models changed when we brought the presence of ASD problems into the equation for predicting ADHD problems and vice versa; this to determine whether identified indicators were generic or specific to these problems. Statistical analyses were performed using SPSS Windows version 16.

## Results

Out of our overall sample (n = 1,816), 348 adolescents met the ASD criterion and 419 fulfilled the ADHD criterion. One hundred and ninety-six met both ASD and ADHD criteria. Descriptive statistics of predictor variables in the ASD and ADHD groups, as well as in the remainder of the total sample, i.e. those who are not in either the ASD or the ADHD group, are presented in Table 2. The Mean of the CSBQ was 5.5 at T1 (SD = 5.6), 5.1 at T2 (SD = 5.8) and 5.0 at T3 (SD = 5.7). The Mean of the CBCL was 3.8 at T1 (SD = 3.1), 2.8 at T2 (SD = 2.7) and 2.5 at T3 (SD = 2.6).

**Table 2:** Scores on early indicators in Autism Spectrum Disorders (ASD) and Attention Deficit/Hyperactivity Disorder (ADHD) groups and the remaining of the sample

Indicators from PCH file	ASD^a^ group n = 348	ADHD^b^ group n = 419	Remaining of the sample n = 1245
	N (%)	N (%)	N (%)
Boys	215 (61.8 %)	253 (60.4 %)	543 (43.6 %)
Low education of mother	141 (40.5 %)	179 (42.7 %)	332 (34.2 %)
Low education of father	110 (31.6 %)	125 (29.8 %)	339 (27.2 %)
Maternal alcohol use during pregnancy	66 (19 %)	94 (22.4 %)	247 (19.8 %)
Maternal smoking during pregnancy	124 (35.6 %)	158 (37.7 %)	365 (29.3 %)
Low birth weight (< 2500 grams)	26 (7.5 %)	24 (5.7 %)	60 (4.8 %)
Birth defects	7 (2.0 %)	7 (1.7 %)	27 (2.2 %)
Neurological abnormalities (age 0–4 yrs.)	15 (4.3 %)	25 (6.0 %)	63 (5.1 %)
Head circumference increase (0–6 mos.)	39 (11.2 %)	35 (8.4 %)	94 (7.6 %)
Head circumference increase (6–12 mos.)	27 (7.8 %)	25 (6.0 %)	76 (6.1 %)
Low BMI at age 2	45 (12.9 %)	44 (10.5 %)	143 (11.5 %)
Low BMI at age 3	3 (0.9 %)	8 (1.91 %)	13 (1.0 %)
Low BMI at age 4	2 (0.6 %)	1 (0.2 %)	15 (1.2 %)
Van Wiechen Scheme (VWS): gross motor skills (age 1–15 mos.)	72 (20.7 %)	59 (14.1 %)	261 (21.0 %)
VWS: fine motor skills and adaptation (age 1–15 mos.)	29 (8.4 %)	26 (6.2 %)	83 (6.7 %)
VWS: communication and social behavior (age 1–15 mos.)	30 (8.6 %)	36 (8.6 %)	81 (6.5 %)
Language difficulties (age 1.5–4 yrs.)	32 (9.2 %)	30 (7.2 %)	74 (5.9 %)
Psychomotor difficulties (age 1.5–4 yrs.)	25 (7.2 %)	19 (4.5 %)	44 (3.5 %)
Sleep problems (age 1.5–4 yrs.)	105 (30.2 %)	126 (30.1 %)	287 (23.1 %)
Problems with eating (age 1.5–4 yrs.)	196 (56.3 %)	232 (55.4 %)	604 (48.5 %)
Social problems in behavior (age 1.5–4 yrs.)	38 (10.9 %)	35 (8.4 %)	79 (6.3 %)
Attention hyperactivity problems (1.5–4 yrs.)	133 (38.2 %)	203 (48.4 %)	421 (33.8 %)
Externalizing problems (age 1.5–4 yrs.)	146 (42.0 %)	190 (45.3 %)	446 (35.8 %)
Internalizing problems (age 1.5–4 yrs.)	66 (19.0 %)	64 (15.3 %)	217 (17.4 %)
Absence of parent-reported positive behavior (age 1.5–4 yrs.)	176 (50.6 %)	206 (49.2 %)	507 (40.7 %)

Table 3 gives the crude and multivariate odds ratios (OR), and the 95 % confidence intervals, on the predictors for ASD and ADHD problems on the CSBQ and the CBCL. Male gender, a low level of education of the mother, a low birth weight, language, psychomotor and eating problems, and social problems (all at toddler age) were identified as significant independent determinants of ASD problems on the CSBQ. A low level of education of the father, maternal smoking during pregnancy, head circumference increase (0–6 months), low BMI at age 2, sleep problems and absence of parent-reported positive behavior did not contribute to the model as independent predictors in multivariate analyses. Controlling for ADHD problems in the ASD group left the model virtually unchanged, only a low level of education of the mother dropped below significance levels, with small reductions in OR. The OR for the presence of ADHD problems was substantial (Table 3).

**Table 3:** Associations of Preventive Child Healthcare indicators with Autism Spectrum Disorders (ASD) and Attention Deficit/Hyperactivity Disorder (ADHD) problems: odds ratios (OR) and 95 % confidence intervals (CI)

	ASD group N = 348			ADHD group N = 419		
	OR (crude) 95 % CI	OR (adj) ^a^ 95 % CI	OR (adj) ^b^ 95 % CI	OR (crude) 95 % CI	OR (adj) ^a^ 95 % CI	OR (adj) ^b^ 95 % CI
Boys	**1.71 (1.39**–**2.11)**	**1.87 (1.43**–**2.45)**	**1.63 (1.27**–**2.17)**	**1.75 (1.43**–**2.13)**	**1.74 (1.38**–**2.19)**	**1.41 (1.08**–**1.85)**
Low education of mother	**1.65 (1.28**–**2.13)**	**1.45 (1.12**–**2.11)**	1.40 (0.96–2.03)	**1.74 (1.37**–**2.21)**	**1.52 (1.15**–**2.01)**	**1.45 (1.01**–**2.08)**
Low education of father	**1.48 (1.15**–**1.90)**			**1.59 (1.25**–**2.02)**		
Maternal alcohol use during pregnancy	0.96 (0.74–1.24)			1.10 (0.86–1.40)		
Maternal smoking during pregnancy	**1.24 (1.01**–**1.54)**			**1.60 (1.30**–**1.96)**	**1.36 (1.07**–**1.73)**	1.31 (0.99–1.75)
Low birth weight	**1.67 (1.05**–**2.67)**	**1.93 (1.14**–**3.27)**	**1.95 (1.10**–**3.48)**	1.13 (0.70–1.82)		
Birth defects	0.97 (0.42–2.24)			0.75 (0.33–1.72)		
Neurological abnormalities	0.78 (0.45–1.38)			1.22 (0.76–1.96)		
Head circumference increase (0–6 mos.)	**1.50 (1.04**–**2.26)**			0.91 (0.61–1.35)		
Head circumference increase (6–12 mos.)	1.38 (0.88–2.12)			1.07 (0.50–2.31)		
Low BMI at age 2	**1.54 (1.10**–**2.20)**			1.03 (0.74–1.44)		
Low BMI at age 3	1.07 (1.01–1.14)			2.59 (0.94–7.13)		
Low BMI at age 4	0.66 (0.13–3.48)			0.24 (0.03–2.05)		
Van Wiechen Scheme gross motor skills	0.94 (0.70–1.25)			**0.73 (0.61**–**0.88)**	**0.59 (0.44**–**0.80)**	**0.60 (0.42**–**0.87)**
VWS fine motor skills	1.30 (0.85–2.01)			0.88 (0.56–1.38)		
VWS communication	1.27 (0.83–1.94)			1.28 (0.86–1.91)		
Language difficulties	**1.61 (1.05**–**2.46)**	**1.75 (1.09**–**2.81)**	**1.71 (1.02**–**2.87)**	1.15 (0.75–1.77)		
(Psycho)motor difficulties	**2.22 (1.35**–**3.65)**	**2.41 (1.38**–**4.17)**	**2.60 (1.42**–**4.76)**	1.16 (0.68–1.99)		
Sleep problems	**1.37 (1.06**–**1.78)**			**1.31 (1.05**–**1.63)**		
Problems with eating	**1.33 (1.05**–**1.69)**	**1.34 (1.02**–**1.73)**	**1.28 (1.02**–**2.87)**	**1.38 (1.08**–**1.76)**		
Social problems in behavior	**1.84 (1.23**–**2.73)**	**1.65 (1.04**–**2.60)**	**1.73 (1.06**–**2.84)**	1.25 (0.83–1.88)		
Attention hyperactivity problems	1.10 (0.86–1.40)			**1.95 (1.56**–**2,43)**	**1.71 (1.35**–**2.16)**	**2.04 (1.55**–**2.69)**
Externalizing problems	1.22 (0.96–1.55)			**1.47 (1.18**–**1.84)**		
Internalizing problems	1.16 (0.86–1.56)			0.82 (0.61–1.11)		
Absence of parent-reported positive behavior	**1.43 (1.13**–**1.81)**			**1.36 (1.09**–**1.70)**	**1.36 (1.06**–**1.75)**	1.25 (0.95–1.64)
ADHD or ASD problems in adolescence			**6.48 (4.85**–**8.66)**			**6.85 (5.10**–**9.20)**

**Criterion** *p* < 0.2 for inclusion in multiple logistic regression model

^a^adj = adjusted for all other variables which are included in the multiple logistic regression model

^b^adj = adjusted for all other variables which are included in the multiple logistic regressionmodel including ADHD problems in ASD and ASD problems in ADHD groups

For ADHD problems on the CBCL, male gender, a low level of education of the mother, maternal smoking during pregnancy, gross motor skills (during the first year), attention hyperactivity problems, and absence of parent-reported positive behavior (at toddler age) were determinants. A low level of education of the father, sleep problems, problems with eating and externalizing problems did not contribute to the model as independent predictors in multivariate analyses. Controlling for ASD problems in the ADHD group left the model nearly intact; only maternal smoking during pregnancy and the absence of parent-reported positive behavior dropped below significance levels, with small reductions in ORs. The OR for the presence of ASD problems was substantial (Table 3).

## Discussion

This study was the first to use early childhood findings as registered by community pediatric services to predict ASD and ADHD problems in adolescents in a large longitudinal community-based sample. We identified several early childhood indicators predictive of ASD and ADHD problems. Male gender and low level of education of the mother were generic indicators, while a low birth weight, social behavioral problems, language, and psychomotor and eating problems at toddler age were specific for ASD problems, and maternal smoking during pregnancy, gross motor skills during infancy, and attention hyperactivity problems at toddler age, for ADHD problems.

We found two important results that warrant further comment. Both controlling for ADHD problems in the ASD group and controlling for ASD problems for the ADHD group did leave the model virtually unchanged and vice versa, while the OR for the presence of ASD and ADHD problems was substantial. This indicates that neither risk indicators for ASD nor risk indicators for ADHD can be attributed solely to the shared variance between ADHD and ASD, therefore, early developmental risk indicators are to some extent specific for ASD and ADHD.

A second intriguing result was that early attention and hyperactivity problems were predictive for later ADHD problems but not ASD problems while early social problems in behavior were predictive for later ASD problems but not ADHD problems. In contrast, during adolescence ADHD is predictive for ASD and vice versa (St Pourcain et al. 2011). This finding points to specificity of risk indicators measured very early in development.

Our study adds important information because our approach uses PCH data in the general community which differs from previous studies on early indicators of ASD or ADHD. Previous studies on ASD focused on clinically-referred and selective high-risk children, such as siblings of children with ASD (Goodlin-Jones et al. 2008; Landa and Garrett-Mayer, 2006; Ozonoff et al. 2010). Previous studies about early indicators of ADHD are scarce, and none of them was based on prospectively collected data to a younger age than 2 years (Loe et al. 2008; Smidts and Oosterlaan, 2007). The few population studies on early risk indicators of ASD and ADHD, report similar odds ratios (1.25–3.43) as we found, see St Pourcain, et al. (2011). The prospective and community-based nature of our study thus adds important information to what was already known.

Importantly, none of these studies used data from the PCH services, even though these services routinely follow the physical (diseases and growth) and psychosocial development of all children from birth on, in addition to screening for different disorders. Moreover, PCH services collect and register data according to a highly standardized format, including a number of objective parameters (e.g., birth weight, BMI, head circumference), therefore providing an outstanding setting for studying risk indicators in relatively unbiased general population samples.

Given our focus on the general community, it is important to note that we identified largely the same early indicators as have been reported for referred and selected high risk ASD and ADHD samples, i.e. pre- and perinatal factors (Smidts and Oosterlaan, 2007), early behavioral signs associated with ASD including impairments or delay in social-communicative development (Hartley et al. 2008; Ozonoff et al. 2010), early behavioral signs such as hyperactive-impulsive and inattentive temperamental features (Hirshfeld-Becker et al. 2002; Smidts and Oosterlaan, 2007), motor, language and speech development (Hagberg et al. 2010; Landa and Garrett-Mayer, 2006; Loe et al. 2008), atypicalities in regulation functions related to eating (Werner and Dawson, 2005) and family adversity (Lara et al. 2009). This suggests that findings from selected samples may be generalized to community-based samples and vice versa, and emphasizes how useful the PCH setting can be for identifying and monitoring both mild and clinical ASD and ADHD.

The major strength of our study is the uniqueness of its design: its prospective nature, spanning many years from birth on, and its embedding in a routine PCH setting. Another strength is that we studied ASD and ADHD problems in tandem, therefore the first to report on generic and specific early childhood indicators. A further strength is our use of multiple indicators of varying types, as previous ASD or ADHD studies only used subsets of indicators. Recent overviews have shown that in most prognostic studies single rather than multiple indicators are investigated, but that multiple indicators provide better models (Burton and Altman, 2004; Riley et al. 2003). A final strength of this study is that the identification of ASD and ADHD problems was based on three ratings, each two-and-a-half years apart, thus reducing measurement error. Also, TRAILS constitutes a large sample with a high response rate, and we retrieved PCH files for the large majority of the participants.

Our study also has limitations, but insofar as these may have affected our results, they will all lead to underestimation of the predictive power of the PCH findings. First, children may have received effective treatment for their developmental problems between the ages of 4 and 11. Second, children in our sample had a lower mean score on ASD and ADHD problems than the remaining of the sample for whom PCH files could not be retrieved*,* and also, as a result of the design of TRAILS, there were no cases of ASD with severe mental retardation in our study, while ASD problems are common in these children (de Bildt et al. 2003; Oeseburg et al. 2010). A related third issue is that some highly predictive early risk indicators are low prevalent and thus were not identified as indicators due to their low prevalence. This may, for instance, concern birth defects. Moreover, for some early risk indicators we lacked detailed information, for example, we knew if the mother smoked during pregnancy but not the daily dose of cigarettes. A fourth limitation is that the used questionnaires were not diagnostic tools performed by trained clinicians, and that we neither used semi-structured interviews. Previous studies, in particular where ASD is concerned, used (semi-)structured interviews which are less susceptible to measurement error and informant bias. Our questionnaires may have introduced additional measurement error due to interpretation problems by the parents. However, gold standard clinician-based interviews such as the ADI (Rutter et al., 2003), the K-SADS (Kaufman et al. 1997) and the PACS (Helverschou et al., 2009) are designed to pick up on only the severe cases within ASD, and ADHD patients. Considering the purpose of the current study they were of no use in our general population sample. Note that part of the measurement error was removed by repeated measurement of ASD and ADHD problems.

In conclusion, the community pediatric services are a useful setting for both research on early predictors of ASD and ADHD problems in general population samples and for the early detection of children at risk in routine care. From early childhood PCH-registered routine data of a large community based sample we identified largely the same early indicators of ASD and ADHD problems as have been reported for clinically referred ASD and ADHD samples. This shows that routine PCH can identify children at risk of both mild and clinical ASD and ADHD in early childhood. The community pediatric services may also play an important role in close monitoring of children identified in such a way, by administering in depth diagnostic instruments to further qualify their symptoms, and by providing early treatment if needed. Notwithstanding relatively modest differences, this may have a rather huge public health impact as these services cover the full population of children (Rose, 1992).

## Abbreviations

ADHD: Attention Deficit/Hyperactivity Disorder
ASD: Autism Spectrum Disorders
CBCL: Child Behavior Checklist
CSBQ: Children’s Social Behavior Questionnaire
OR: Odds Ratios
PCH: Preventive Child Healthcare
TRAILS: Tracking Adolescents’ Individual Lives Survey
